# What does anisotropy measure? Insights from increased and decreased anisotropy in selective fiber tracts in schizophrenia

**DOI:** 10.3389/fnint.2013.00009

**Published:** 2013-03-11

**Authors:** L. M. Alba-Ferrara, Gabriel A. de Erausquin

**Affiliations:** Roskamp Laboratory of Brain Development, Modulation and Repair, Morsani College of Medicine, University of South FloridaFL, USA

**Keywords:** dopamine, Parkinsonism, schizophrenia, DTI, white matter, myelination

## Abstract

Schizophrenia is a common, severe, and chronically disabling mental illness of unknown cause. Recent MRI studies have focused attention on white matter abnormalities in schizophrenia using diffusion tensor imaging (DTI). Indices commonly derived from DTI include (1) mean diffusivity, independent of direction, (2) fractional anisotropy (FA) or relative anisotropy (RA), (3) axial diffusivity, and (4) radial diffusivity. In cerebral white matter, contributions to these indices come from fiber arrangements, degree of myelination, and axonal integrity. Relatively pure deficits in myelin result in a modest increase in radial diffusivity, without affecting axial diffusivity and with preservation of anisotropy. Although schizophrenia is not characterized by gross abnormalities of white matter, it does involve a profound dysregulation of myelin-associated gene expression, reductions in oligodendrocyte numbers, and marked abnormalities in the ultrastructure of myelin sheaths. Since each oligodendrocyte myelinates as many as 40 axon segments, changes in the number of oligodendrocytes (OLG), and/or in the integrity of myelin sheaths, and/or axoglial contacts can have a profound impact on signal propagation and the integrity of neuronal circuits. Whereas a number of studies have revealed inconsistent decreases in anisotropy in schizophrenia, we and others have found increased FA in key subcortical tracts associated with the circuits underlying symptom generation in schizophrenia. We review data revealing increased anisotropy in dopaminergic tracts in the mesencephalon of schizophrenics and their unaffected relatives, and discuss the possible biological underpinnings and physiological significance of this finding.

## Introduction: what does DTI measure?

Diffusion MRI allows to estimates brain fiber structures using water diffusion properties as a probe (Mori, 2007). In an unstructured space, water molecules diffuse freely in a random (Brownian) manner. Such random motion is called *isotropic* reflecting the relatively equal probability of each molecule to move in any given direction. On the other hand, in structured spaces, the probability of water diffusion is constrained in some directions but unconstrained in others. For example, in the case of brain tissue, water molecules diffuse more freely along the axon but are relatively constrained from escaping it or moving across the walls of axons. This coherent directionality is therefore called *anisotropic*. By calculating the diffusivity along multiple directions, the diffusion tensor may be calculated and it becomes possible to estimate the orientation of axon bundles (Mori, 2007).

This assumption that anisotropy in white matter is caused by cellular structures delimiting free water diffusion is reasonable at face value. Thus, the axon itself (a tubular, relatively rigid structure) may be sufficient to generate anisotropy in white matter. Additional limitations to molecular motion come from myelination, as multiple myelin sheets create a compact lipidic cover that isolates the axon. Experimental testing in an animal model was used to compare fractional anisotropy (FA) of normal and demyelinated mice, and found a 20% reduction of FA in the latter (Song et al., 2002). Thus, although myelination contributes to FA, other sources, including the axon itself, seem to provide the greatest FA. Additionally, it is unknown whether barriers or intermolecular binding affect the water diffusion within the axon, but it is logical to assume so.

In spite of the uneven contributions of axon and myelin to anisotropy, changes to each of these structures cannot be distinguished on the basis of diffusion tensor imaging (DTI) results. To illustrate, a recent investigation in animal models of multiple sclerosis compared mice treated with the drug cuprizone, which produces inflammation and demyelination without damaging the axons themselves, against mice treated with a combination of cuprizone and a peptide which leads to demyelination as well as axonal damage. Perhaps unsurprisingly, the results revealed that FA values cannot distinguish between the demyelination group and the demyelination plus axonal damage group (Boretius et al., 2012). Combination of DTI with histological techniques may facilitate the interpretation of underlying pathology in white matter diseases.

Thus, although diffusion anisotropy theoretically reflects microscopic anatomy, spatial resolution obtained by MR-DTI remains at the macroscopic level (a typical voxel size is around 2 by 3 mm). Consequently, discrepancies between levels, that is, the microscopic level which underlies anisotropy and the macroscopic level observed with this technique, are to be expected and inferences regarding the former on the basis of the latter should be made with caution. A particularly challenging illustration is the identification of crossing fibers. In this situation, a single voxel may be composed by fiber populations with different spatial orientation resulting in an average increase in FA, which in such a case would not be due to changes in axonal or myelin structure. Crossing fibers have a remarkable impact in tractography and anisotropy analysis, as an estimated of 90% white matter voxels contain crossing fibers (Jeurissen et al., 2012) leading to the suggestion that in DTI analysis macroscopic factors are preponderant and may override microscopic factors (Mori, 2007). For the previously exposed reasons, FA is not always a reliable marker of white matter integrity and convergent findings from different techniques should be used to interpret the results. Indeed, an alternative method has recently been developed that may overcome some of the limitations of FA analysis. The new method models the effect of spin motion on the MRI signal to extract information about the microstructure from the motion of the spins, allowing to represents the motion properties in an asymmetric, unconstrained, unrestricted fashion (Ozcan, 2010). This approach may shed new light on the changes at the intersection of crossing fibers pathways and within isosurfaces of gray matter (Ozcan, 2010).

## Oligodendrocytes in schizophrenia

White matter abnormalities have been proposed to be central to the pathophysiology of schizophrenia (Haroutunian and Davis, 2007; Takahashi et al., 2011), but the neurobiological substrates of these abnormalities remain elusive. Recent evidence suggests that dysfunction in myelination and altered oligodendrocytes (OLG) number and function may contribute to schizophrenia (Flynn et al., 2003; Vostrikov et al., 2007; Katsel et al., 2008), and myelin and fatty-acid biosynthesis dysfunction was reported on post-mortem brain (prefrontal cortex) of schizophrenia utilizing parallel metabolic and transcriptomics investigations (Tkachev et al., 2007). The latter study revealed that N-acetylaspartarte, a source of acetyl groups which are incorporated into myelin, is deregulated in schizophrenia possibly underlying reductions in white mater volume.

Focus on altered central nervous system myelination in schizophrenia has led to a number of studies implicating oligodendrocyte dysfunction (Uranova et al., 2001, 2004; Tkachev et al., 2007; Konrad and Winterer, 2008). For example, mRNA expression of four oligodendrocyte related genes with variants associated with schizophrenia found that although expression was not reduced, patients carrying risk alleles had lower transcript levels (Mitkus et al., 2008). Likewise, expression of a variety of oligodendrocyte genes has been found to be altered particularly the temporal lobe (Katsel et al., 2005). Oligodendrocyte pathology seems to be closely related to MRI-detected white matter abnormalities in schizophrenia (Segal et al., 2007) although there is no direct evidence of a causal link between them.

Several recent studies attempt to directly demonstrate a link between genetic variation and white matter integrity (Braskie et al., 2012; Felsky et al., 2012; Prata et al., 2012). Thus, a single nucleotide polymorphism (rs1059004) in the gene for the transcription factor OLIG2 (necessary for oligodendrocyte generation) has an allele associated with reduced white matter integrity, measured with FA, in the corona radiata bilaterally in healthy volunteers (Prata et al., 2012). Notably, genetic variation within the OLIG2 gene (including the allele just referenced) is associated with schizophrenia in at least two samples (Georgieva et al., 2006; Huang et al., 2008).

Similarly, genetic variation in the NTRK1 gene, which is associated with nervous system development and myelination, also predicts FA in healthy volunteers (Braskie et al., 2012). Interestingly, diffusion perpendicular to the axon fiber but not diffusion parallel to the fiber was directly correlated to FA in this sample. Such finding has implications for the molecular underpinning of the DTI measure, as diffusion perpendicular to the axonal tracts is thought to reflect lack of myelin and increased permeability instead diffusivity parallel to the axonal tracts may reflect axonal integrity (Song et al., 2002). Again this finding is in line with investigations showing an association between variation in NTRK1 and genetic risk for schizophrenia (van Schijndel et al., 2009, 2011).

Finally, post-mortem studies of anterior frontal cortex shown reduced protein expression of two oligodendrocyte-associated proteins in schizophrenia (2′,3′-cyclic nucleotide 3′-phosphodiesterase and myelin-associated glycoprotein) (Flynn et al., 2003), but a recent paper assessing genetic variation within the gene for the latter of these proteins and MRI parameters found no association to white matter integrity (Felsky et al., 2012). Further studies linking genetic variations in schizophrenia with white matter abnormalities are anticipated.

## Fiber tracts integrity in schizophrenia

In the preceding paragraphs we have discussed data suggesting dysfunction of white matter components and in diffusion weighted MRI in schizophrenia without a particular attention to specific white matter tracts. In general, FA is globally decreased (Douaud et al., 2007; Kubicki et al., 2007), but there are some tracts which present increased FA in schizophrenia patients in comparison to controls (Kubicki et al., 2007). Also, although low FA is usually associated to poor cognitive performance in healthy as well as in some clinical populations (Turken et al., 2008), in schizophrenia this is not always the case (Okugawa et al., 2006). Since an exhaustive review of the white matter changes in schizophrenia is beyond the scope of this paper, over the next few paragraphs we will focus on those changes that have been shown to correlate specifically with models of symptom generation which may help understand the relationship between anisotropy and functional changes. We will pay particular attention in our comments to studies of subcortical white matter (Table 1) which have not been specifically discussed in currently published reviews even though several original publications have become available (see below).

**Table 1:** **Fractional anisotropy in subcortical white matter of subjects with schizophrenia**.

**White matter tract**	**Fractional anisotropy**	**References**
Superior longitudinal fasciculus	Increased in ah compared to non-ah patients	Seok et al., 2007; Shergill et al., 2007
Arcuate fasciculus	Increased in AH compared to non-ah patients	Hubl et al., 2004; Rotarska-Jagiela et al., 2009
Corpus callosum	Increased in hallucinators compared to HC	Hubl et al., 2004
Substantia nigra	Increased in compared to controls	Toranzo et al., 2011
Ventral tegmental area	Increased in compared to controls	Toranzo et al., 2011
Inferior fronto-occipital fasciculus (bilateral)	Reduced in patients compared to HC	Rotarska-Jagiela et al., 2009; Walther et al., 2011
Anterior corona radiata (right)	Reduced in patients compared to HC	Walther et al., 2011
Uncinate fasciculus (left)	Reduced in patients compared to HC	Walther et al., 2011
Posterior corona radiata	Reduced in patients compared to HC	Cui et al., 2011
Whole brain	Reduced in patients compared to HC	Douaud et al., 2007

Schizophrenia is reportedly associated with reduced FA in frontal regions, which correlates with cognitive and motor deficits in patients (Walther et al., 2011). In addition, better performance on attention and executive function tasks was associated with higher levels of FA in task-relevant regions in subjects with schizophrenia (Lim et al., 2006). But contrary to the excessively simplistic postulate that more FA is always better, patients who hear conversing hallucinations have increased FA in interhemispheric auditory fibers compared to patients without this symptom or, to a lesser extent, to healthy controls (Mulert et al., 2012). Moreover, higher FA in fibers connecting temporal regions (such as the arcuate fasciculus or the superior longitudinal fasciculus) was found to be associated with increased severity of hallucinations in schizophrenia patients (Hubl et al., 2004; Seok et al., 2007; Shergill et al., 2007; Rotarska-Jagiela et al., 2009).

The arcuate fasciculus connects the posterior temporal gyrus with the inferior frontal gyrus which underlies speech and vocal processing. Increased FA in the arcuate fasciculus bilaterally is likely to contribute to the pathophysiology underlying hallucinations in schizophrenia (Alba-Ferrara et al., 2012); it combines with reduced functional connectivity between the posterior superior temporal gyrus and the anterior cingulate cortex resulting in difficulties to judge whether verbal stimuli are originated in the brain or come from an external source (Alba-Ferrara et al., 2012). As a result of such changes, persons with schizophrenia would misidentify self-generated auditory objects as coming from external sources (Mechelli et al., 2007), causing or contributing to hallucinations. Alternatively, but not necessarily in contradiction, diffusion abnormalities may decrease speed of axonal transmission speed resulting in neural timing abnormalities. Indeed, schizophrenia patients experience time-delayed corollary discharges to self-generated auditory stimuli, resulting in aberrant suppression of the sensory consequences of self-generated actions (Whitford et al., 2011). Thus, schizophrenia patients have a combination of increased and decreased structural and functional connectivity in specific brain networks, and it is likely that different clinical profiles may explain differences in the reported findings on DTI studies.

Although a vast majority of DTI studies in schizophrenia have focused in the white matter tracts connecting between cortical structures, subcortical structures have recently started to draw the focus of attention. Connectivity of cortico-subcortical tracts has been found to be reduced (as measured by FA) in the posterior corona radiata of persons with schizophrenia in one study (Cui et al., 2011), but not in another (Zhang et al., 2012). Corona radiata contains reciprocal connecting fibers from the thalamus to the cerebral cortex and descending fibers from the frontoparietal cortex to the basal ganglia. Curiously, a strong negative correlation between motor activity and decreased FA was reported in schizophrenia patients (Walther et al., 2011).

The shift to focusing on subcortical structures is in line with re-emergence of the dopaminergic hypothesis of schizophrenia. In brief, it has been proposed that striatal dopamine hyperfunction may contribute to the positive symptoms of schizophrenia (such as hallucinations or delusions), whereas decreased dopamine availability in the prefrontal cortex may underlie its negative symptoms (such as cognitive impairment, abulia or anhedonia) (de Erausquin et al., 1995; Masciotra et al., 2005; Howes and Kapur, 2009). This hypothesis has received support from a variety of anatomical, functional and experimental sources (Masciotra et al., 2005). In previous studies from our laboratory, an indigenous population of never-treated schizophrenics and their first-degree relatives were identified (Strejilevich et al., 2005; Calvó de Padilla et al., 2006). We recently reported preliminary DTI data on these medication free patients and their relatives focusing on dopaminergic fiber tracts. We found that FA was increased in dopaminergic tracts of both schizophrenia patients and their unaffected first degree relatives, when compared to healthy controls (Toranzo et al., 2011). To the best of our knowledge, these data are the first reported free of the confounding effect of medications on DTI.

Recently, it was proposed that abnormal myelination in frontal regions may result in conduction delays in the efferent copies initiated by willed action. As a consequence, corollary discharges may be generated too late to suppress the sensory consequence of the willed action. Such anachronism would trigger prediction errors mechanisms underpinned by increased midbrain dopaminergic activity (Whitford et al., 2012). Our data is also in line with the idea that overactivation of ascending dopamine pathways may underlie hyperkinetic movements in prodromal schizophrenia (Mittal et al., 2008). Exacerbated activity of the dopamine receptors in the striatal pathway has been found in psychosis (Seeman and Kapur, 2000). It could be thought that such neural overactivity may modulate synaptic maps resulting indeficient axonal pruning (Cohen-Cory, 2002). Deficient axonal pruning may lead to redundant networks which may indicate decreased efficiency in information transmission reflected in increased FA in schizophrenia. Following this line, it has been hypothesized that forming and maintaining the brain's axonal wiring is metabolically costly (Laughlin and Sejnowski, 2003). As it is assumed that the brain attempts to minimize wiring costs, perhaps the schizophrenia brain increased FA is an indicator of poor cost efficiency.

## Conclusions and future directions

To summarize, we have highlighted that in schizophrenia there is an overall reduction of FA globally and some focal increased FA as exemplified in the networks underlying symptoms such as hallucinations and delusions as well as movement disorders. Whereas the diminished structural connectivity is thought to result in the negative features of schizophrenia, exacerbated connectivity of certain brain regions are thought to reflect excessive salience and or focus on irrelevant stimuli as in the case of hallucinations (Toranzo et al., 2011), or perhaps it might reflect aberrant axonal pruning through neurodevelopment leading to maintenance of inefficient/redundant neural networks as in the case of dopaminergic projections. Generalized aberrant connectivity, reflecting both increases and decreases in FA, could point to either a diffuse dysregulation of neural dynamics or possible compensatory changes in response to primary deficits. Our own preliminary data shows that first degree relatives of schizophrenia patients present FA values in between healthy controls and patients we are inclined to consider the first option mode likely (Toranzo et al., 2011). A cautionary word is necessary nonetheless about the limitations of FA signal measurements to measure axonal integrity as discussed in the first half of this review (Ozcan, 2010; Jeurissen et al., 2012), which emphasizes the precarious nature of present interpretations and the acute need for further experimental data and new analytical techniques.

### Conflict of interest statement

The authors declare that the research was conducted in the absence of any commercial or financial relationships that could be construed as a potential conflict of interest.
